# Supplementary material for: Bacteria evolve macroscopic multicellularity by the genetic assimilation of phenotypically plastic cell clustering
Source: Nat Commun. 2023 Jun 15;14:3555. doi: 10.1038/s41467-023-39320-9 (PMC10272148; doi:10.1038/s41467-023-39320-9)
Supplement: Supplementary file 1 — Supplementary Information [file 41467_2023_39320_MOESM1_ESM.pdf]

Supplementary Information

for

Bacteria evolve macroscopic multicellularity by the genetic assimilation of phenotypically plastic cell clustering

Yashraj Chavhan[1,*], Sutirth Dey[2] & Peter A. Lind[1,3*]

*Correspondence to:
Yashraj Chavhan (yashraj.chavhan@umu.se)
or
Peter A. Lind (peter.lind@umu.se)

Affiliations
[1]Department of Molecular Biology, Umeå University, Umeå, Sweden
[2]Indian Institute of Science Education and Research (IISER) Pune, Pune, India
[3]Umeå Centre for Microbial Research, Umeå University, Umeå, Sweden

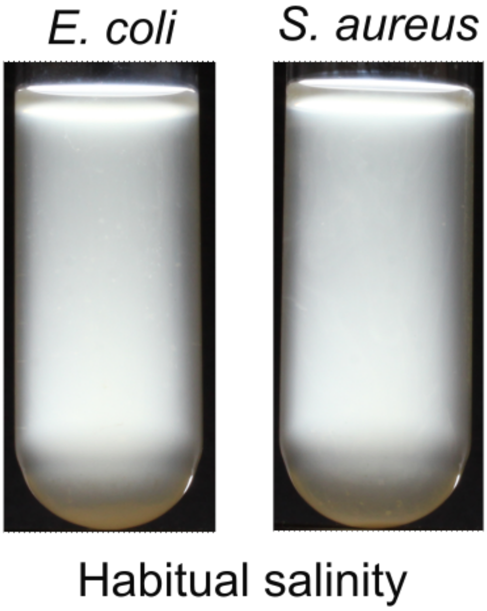

Fig. S1. Both *E. coli* and *S. aureus* clusters disintegrate and grow planktonically upon being transferred to the habitual salinity environment (incubation period: 24 h).

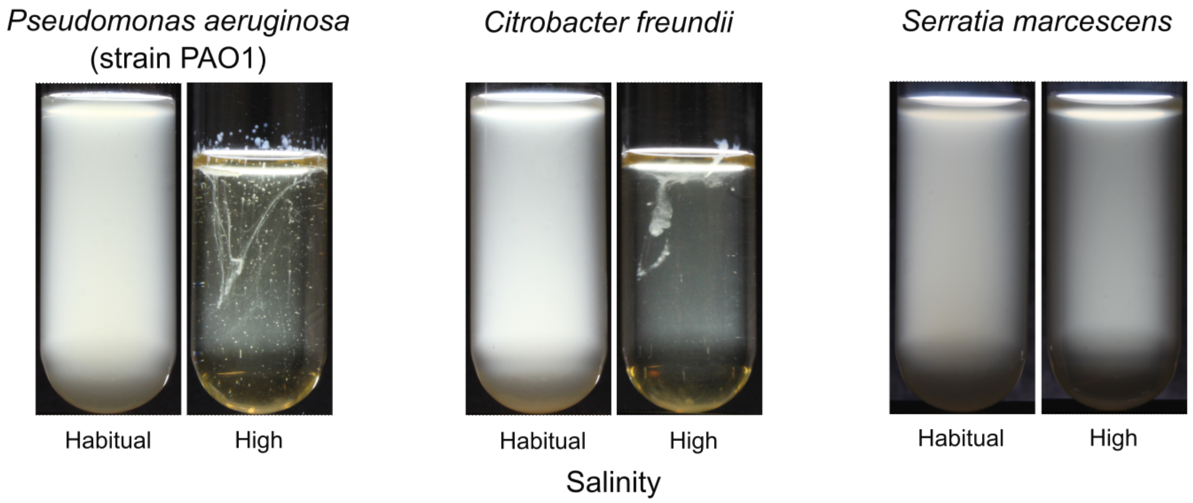

Fig. S2. Phenotypic plasticity of bacterial growth under habitual versus high salinity. Incubation times: *Pseudomonas aeruginosa* (strain PAO1): 24 h under habitual salinity, 72 h under high salinity (reduction in volume due to evaporation); *Citrobacter freundii*: 24 h under habitual salinity, 72 h under high salinity (reduction in volume due to evaporation); *Serratia marcescens*: 24 h under both habitual and high salinity.

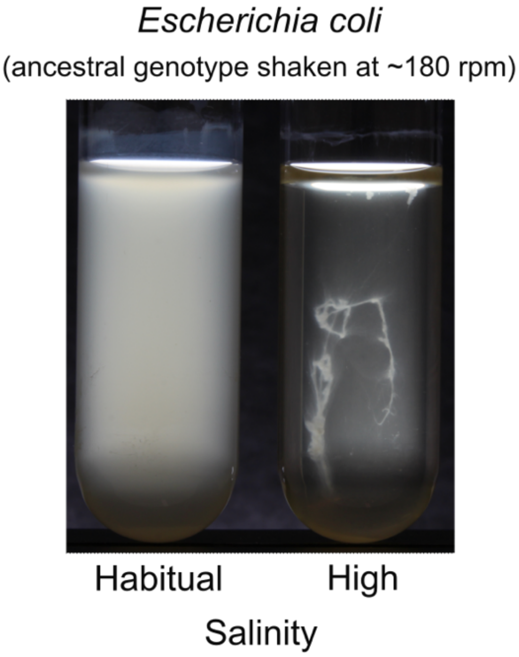

Fig. S3 Phenotypically plastic macroscopic cell clustering exhibited by *E. coli* MG1655 under well mixed conditions in tubes shaken at ~180 rpm (incubation period: 24 h).

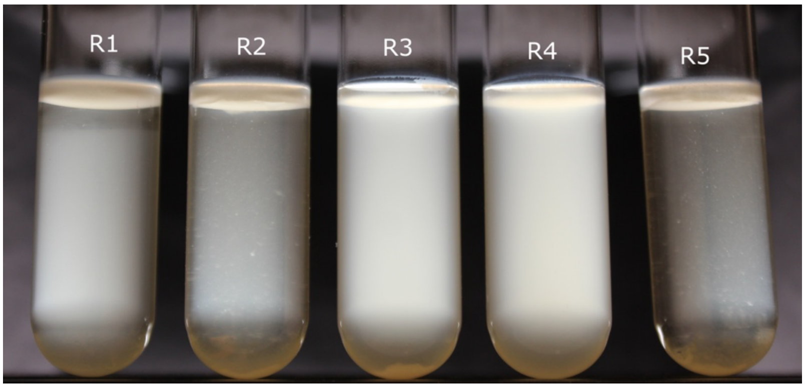

Fig. S4. The R1-R5 clones grown under habitual salinity pictured just prior to external perturbation. R1, R2, R3, and R5 were successful in genetically assimilating macroscopic multicellularity by inherently growing as interface dwelling mats even without environmental induction. Also see Fig. 2b.

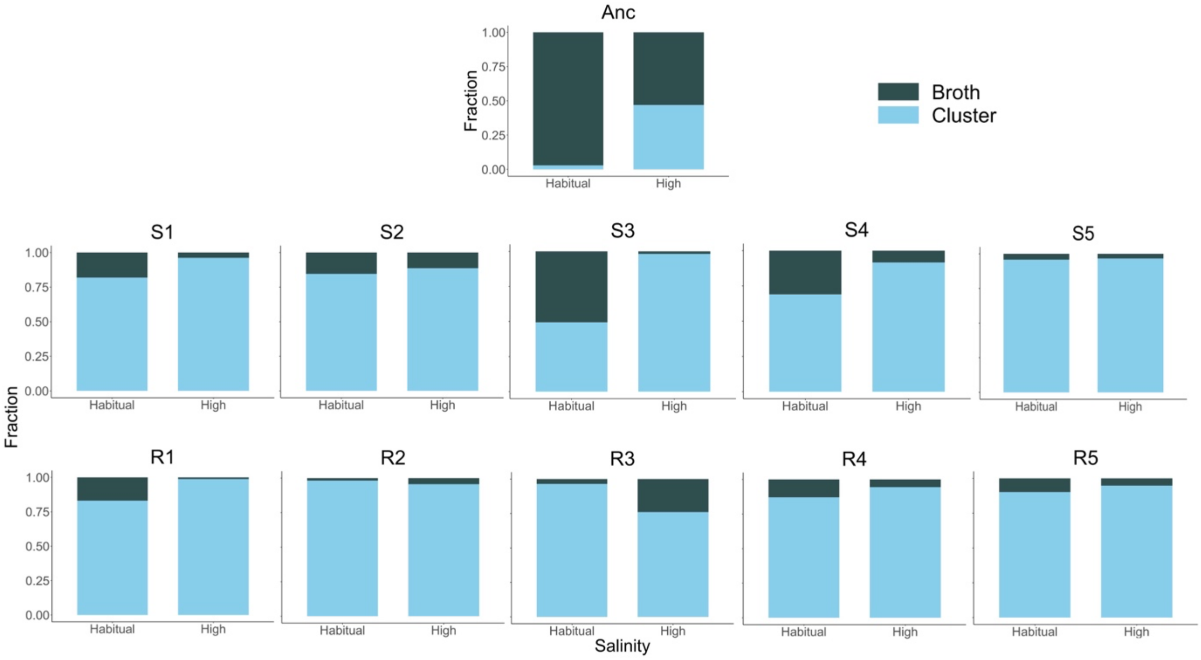

Fig. S5. The relative fractions of viable cells within multicellular clusters and the turbid broth. In both S and R, the majority of viable cells were found within clusters and not in the planktonic broth. Moreover, in both S and R, the fraction of viable cells within clusters was higher than that for the ancestor, under both habitual and high salinity: Two-tailed single sample *t*-tests against the ancestral level: S (habitual salinity): $P = 0.0008$; S (high salinity): $P = 1.2 \times 10^{-5}$; R (habitual salinity): $P = 6.4 \times 10^{-6}$; R (high salinity): $P = 0.0004$. Each plot represents the mean of two independently conducted assays; see the *Source Data* file for the complete dataset and viable colony counts. Also see Fig. S7.

Table S1. Summary of two-tailed *t*-tests (unequal variance across types) on cellular perimeter across habitual and high salinity (N = 40).

Clone	Mean perimeter under habitual salinity (μm)	Mean perimeter under high salinity (μm)	*P* value
Anc	5.78	4.529	0.02
S1	5.132	7.508	0.011
S2	5.887	10.114	0.002
S3	5.385	5.323	0.854
S4	5.209	14.711	0.007
S5	6.048	10.426	0.002
R1	5.747	6.949	0.017
R2	4.618	6.964	2.933×10^{-5}
R3	5.174	15.078	8.592×10^{-5}
R4	4.236	4.522	0.226
R5	5.134	6.094	0.008

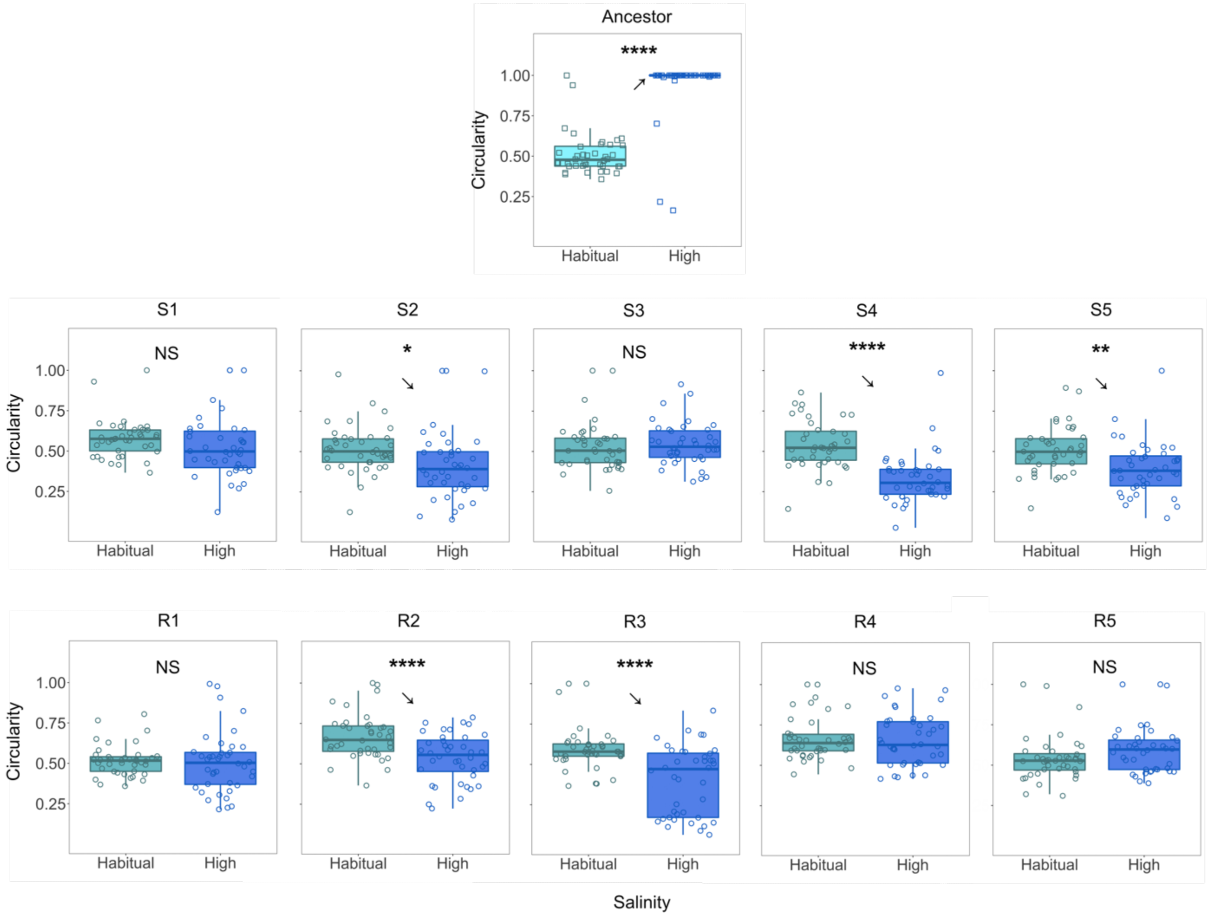

Figure S6. The evolution of phenotypic plasticity in cellular circularity. The arrows point towards the qualitative direction of phenotypic plasticity. The lower and upper box hinges show the 25% and 75% quantiles, respectively; the thick horizontal band represents the median. The lower whisker denotes the smallest observation ≥ lower hinge - 1.5 × interquartile range; the upper whisker represents the largest observation ≤ upper hinge + 1.5 × interquartile range. Two-tailed *t*-tests (unequal variance across types: *: $P \leq 0.05$; **: $P \leq 0.01$; ***: $P \leq 0.001$; ****: $P \leq 0.0001$. See Table S1 for statistical details (exact *P* values). All the raw data are provided in the *Source Data* file.

Table S2. Summary of two-tailed *t*-tests (unequal variance across types) on cellular circularity across habitual and high salinity (N = 40).

Clone	Mean circularity under habitual salinity	Mean circularity under high salinity	*P* value
Anc	0.51	0.951	2.110×10^{-19}
S1	0.576	0.528	0.19
S2	0.514	0.419	0.026
S3	0.53	0.545	0.629
S4	0.54	0.334	5.566×10^{-8}
S5	0.508	0.393	0.002
R1	0.518	0.504	0.673
R2	0.667	0.538	1.357×10^{-4}
R3	0.598	0.4	5.960×10^{-6}
R4	0.666	0.655	0.731
R5	0.543	0.597	0.11

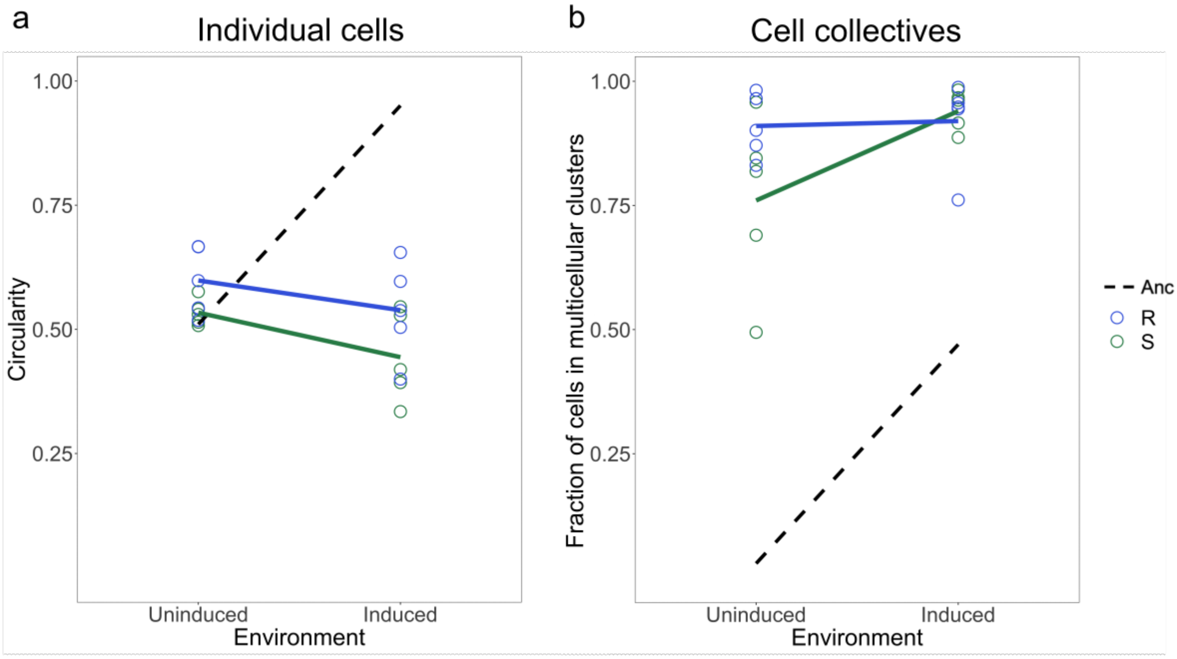

Fig. S7. The evolution of plastic phenotypes in opposite directions at two distinct levels of biological organization. The dashed black lines represent ancestral reaction norms. The thick blue and green lines represent the reaction norms of the R and S lines, respectively. **(a)** At the level of individual cells, the evolved cell circularity was relatively closer to the uninduced ancestral cell circularity. Each data point represents an independently evolving replicate population and is derived from the average of 40 distinct cell circularity values (see Fig. S6 for details and statistical tests). Thus, at the level of individual cells, the evolved phenotypes under both high and habitual salinity matched the uninduced ancestral phenotype. **(b)** At the level of cell collectives, the fraction of evolved cells found within multicellular clusters was relatively closer to the induced ancestral value (as compared to the uninduced value). Both R and S showed genetically assimilated cell clustering (two-tailed *t*-tests (unequal variance across types): $P = 0.852$ for R lines in uninduced vs. induced environments; $P = 0.083$ for S lines in uninduced vs. induced environments). Put differently, at the level of cell collectives, the evolved and assimilated phenotype matched the induced ancestral phenotype more closely than the uninduced phenotype. See Fig. S5 for details. All the raw data are provided in the *Source Data* file.

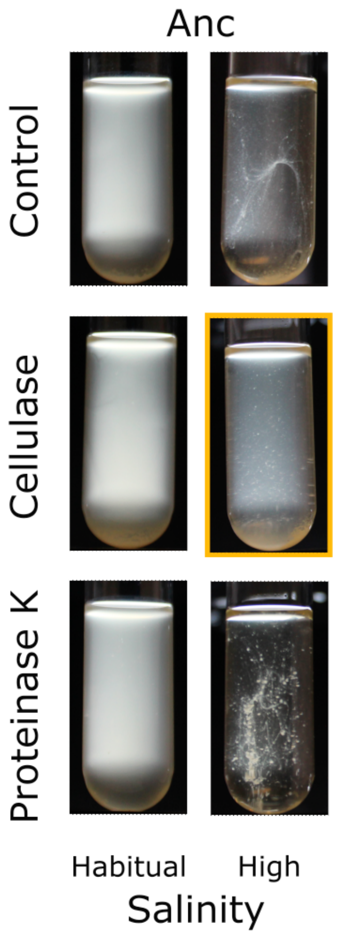

Fig. S8. The development of macroscopic clusters in the ancestor upon incubation with hydrolytic enzymes. Red boxes represent complete inhibition of macroscopic clustering; yellow boxes represent a reduction in or incomplete inhibition of macroscopic clustering. There was no macroscopic clustering under high salinity. Proteinase K did not result in the reduction of macroscopic clustering under high salinity. While cellulase could not inhibit macroscopic clustering completely, it prevented the formation of elongated clusters under high salinity.

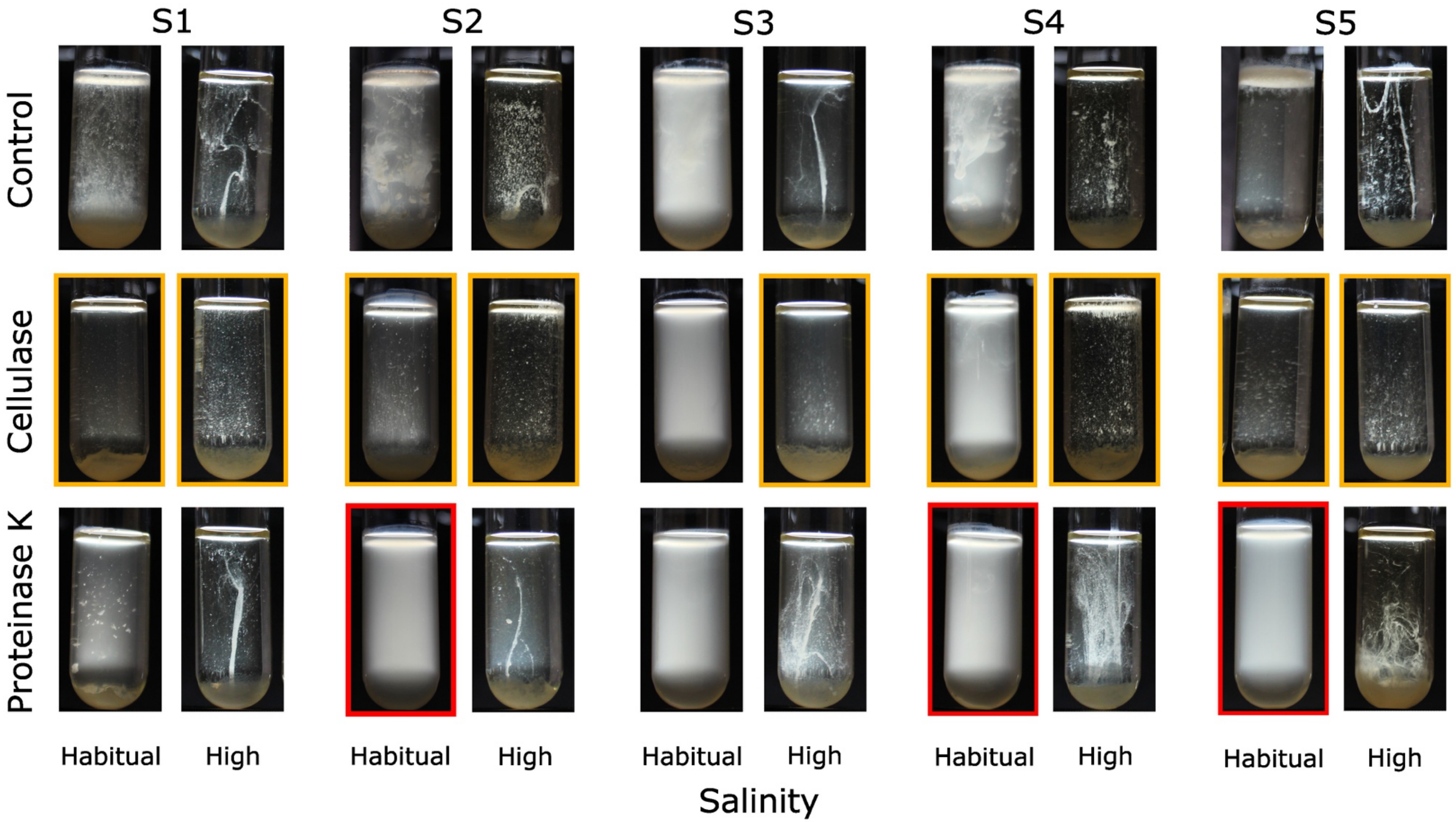

Fig. S9. The development of macroscopic clusters in the S clones upon incubation with hydrolytic enzymes. Red boxes represent complete inhibition of macroscopic clustering; yellow boxes represent a reduction in or incomplete inhibition of macroscopic clustering. Proteinase K inhibited clustering under habitual salinity in 3/5 clones, but resulted in relatively thicker clusters under high salinity in 4/5 clones. Although cellulase failed to inhibit macroscopic clustering completely, it prevented the formation of elongated clusters under high salinity (5/5 clones) and reduced the macroscopic clusters at the air-liquid interface in 4/5 clones. Taken together, the S clones showed qualitatively different clustering glues in the presence and absence of environmental induction.

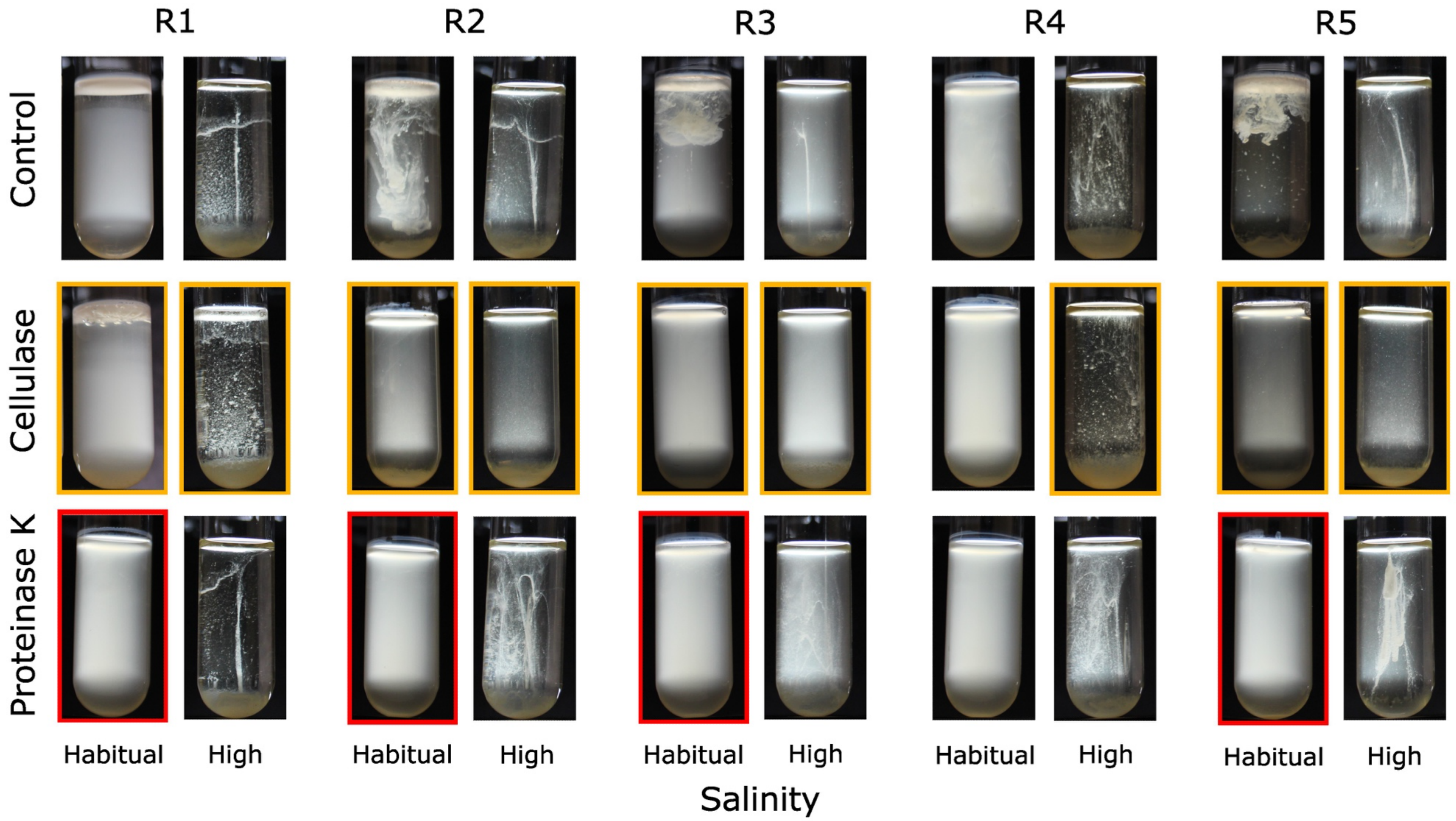

Fig. S10. The development of macroscopic clusters in the R clones upon incubation with hydrolytic enzymes. Red boxes represent complete inhibition of macroscopic clustering; yellow boxes represent a reduction in or incomplete inhibition of macroscopic clustering. Proteinase K inhibited clustering under habitual salinity in 4/5 clones, but resulted in relatively thicker clusters under high salinity in 4/5 clones. Although cellulase failed to inhibit macroscopic clustering completely, it prevented the formation of elongated clusters under high salinity (5/5 clones) and reduced the macroscopic clusters at the air-liquid interface in 4/5 clones. Thus, the R clones showed qualitatively different clustering glues in the presence and absence of environmental induction.

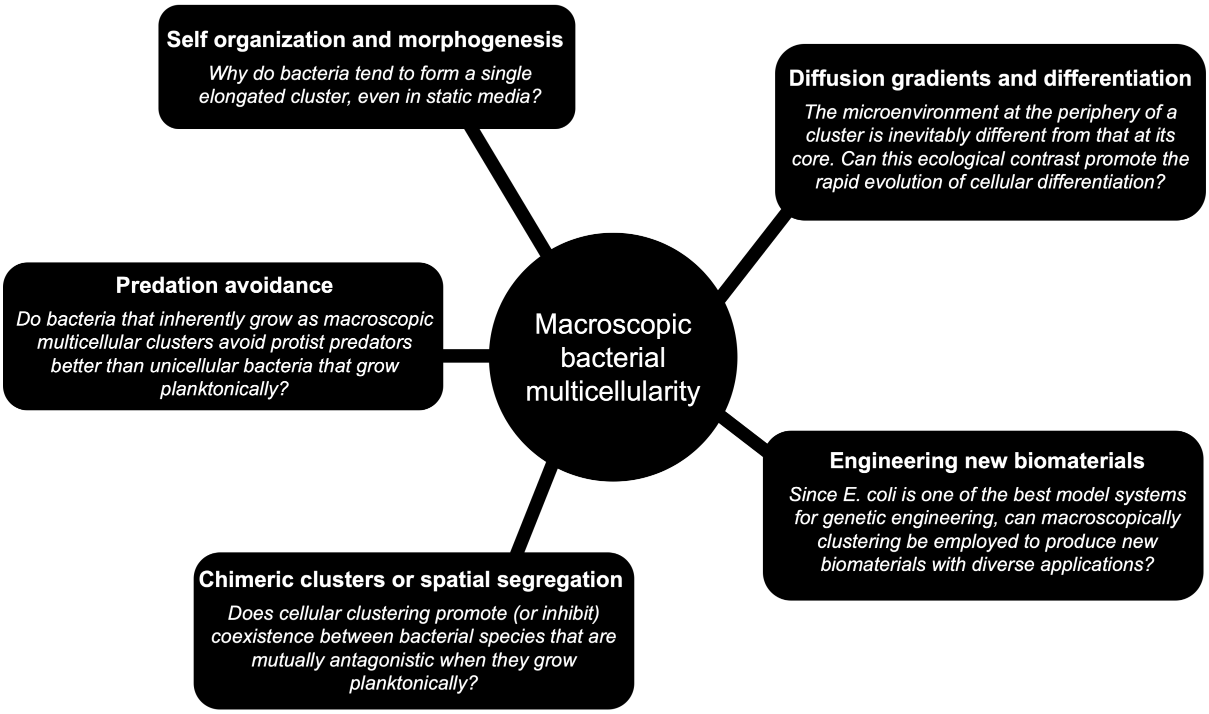

Fig. S11. Some important future research questions and directions likely to spring forth from our results. Taken together, our results should be of interest to a wide variety of scientific fields.
