# Supplementary material for: Bacteria evolve macroscopic multicellularity by the genetic assimilation of phenotypically plastic cell clustering
Source: Nat Commun. 2023 Jun 15;14:3555. doi: 10.1038/s41467-023-39320-9 (PMC10272148; doi:10.1038/s41467-023-39320-9)
Supplement: Supplementary file 3 — Reporting Summary [file 41467_2023_39320_MOESM3_ESM.pdf]

nature portfolio

Corresponding author(s): Yashraj Chavhan (yashraj.chavhan@umu.se), Peter A. Lind (peter.lind@umu.se)

Last updated by author(s): May 9, 2023

Reporting Summary

Nature Portfolio wishes to improve the reproducibility of the work that we publish. This form provides structure for consistency and transparency in reporting. For further information on Nature Portfolio policies, see our Editorial Policies and the Editorial Policy Checklist.

Statistics

For all statistical analyses, confirm that the following items are present in the figure legend, table legend, main text, or Methods section.

n/a	Confirmed	
☐	☒	The exact sample size (n) for each experimental group/condition, given as a discrete number and unit of measurement
☐	☒	A statement on whether measurements were taken from distinct samples or whether the same sample was measured repeatedly
☐	☒	The statistical test(s) used AND whether they are one- or two-sided<br>*Only common tests should be described solely by name; describe more complex techniques in the Methods section.*
☒	☐	A description of all covariates tested
☒	☐	A description of any assumptions or corrections, such as tests of normality and adjustment for multiple comparisons
☐	☒	A full description of the statistical parameters including central tendency (e.g. means) or other basic estimates (e.g. regression coefficient) AND variation (e.g. standard deviation) or associated estimates of uncertainty (e.g. confidence intervals)
☐	☒	For null hypothesis testing, the test statistic (e.g. F, t, r) with confidence intervals, effect sizes, degrees of freedom and P value noted<br>*Give P values as exact values whenever suitable.*
☒	☐	For Bayesian analysis, information on the choice of priors and Markov chain Monte Carlo settings
☒	☐	For hierarchical and complex designs, identification of the appropriate level for tests and full reporting of outcomes
☒	☐	Estimates of effect sizes (e.g. Cohen's d, Pearson's r), indicating how they were calculated

Our web collection on statistics for biologists contains articles on many of the points above.

Software and code

Policy information about availability of computer code

Data collection	We used Persecond for Mac version 1.5 (Flixel Inc. (Toronto, Canada)) for making time-lapse movies.
Data analysis	We used FIJI (ImageJ 1.53) for Mac toanalyze cell shapes by manually tracing the cellular boundaries. We generated the graphs reported in this study using custom scripts run on RStudio (version 2022.02.3 Build 492). We analyzed the sequencing output using the Geneious Prime software for Mac (v2022.0.2). We used BBDuk for trimming the sequencing output data with a quality score < 20.

For manuscripts utilizing custom algorithms or software that are central to the research but not yet described in published literature, software must be made available to editors and reviewers. We strongly encourage code deposition in a community repository (e.g. GitHub). See the Nature Portfolio guidelines for submitting code & software for further information.

Data

Policy information about availability of data

All manuscripts must include a data availability statement. This statement should provide the following information, where applicable:

- Accession codes, unique identifiers, or web links for publicly available datasets
- A description of any restrictions on data availability
- For clinical datasets or third party data, please ensure that the statement adheres to our policy

All relevant data are within the manuscript and its Supplementary Information files. The whole genome sequences reported in this study are available from the NCBI database (accession number: PRJNA880543; https://www.ncbi.nlm.nih.gov/sra/PRJNA880543).

Research involving human participants, their data, or biological material

Policy information about studies with human participants or human data. See also policy information about sex, gender (identity/presentation), and sexual orientation and race, ethnicity and racism.

Reporting on sex and gender	Our study did not involve any human subjects and did not involve any reporting on sex/gender.
Reporting on race, ethnicity, or other socially relevant groupings	Not applicable
Population characteristics	Not applicable
Recruitment	Not applicable
Ethics oversight	Not applicable

Note that full information on the approval of the study protocol must also be provided in the manuscript.

Field-specific reporting

Please select the one below that is the best fit for your research. If you are not sure, read the appropriate sections before making your selection.

☐ Life sciences ☐ Behavioural & social sciences ☒ Ecological, evolutionary & environmental sciences

For a reference copy of the document with all sections, see nature.com/documents/nr-reporting-summary-flat.pdf

Ecological, evolutionary & environmental sciences study design

All studies must disclose on these points even when the disclosure is negative.

Study description	We grew different bacterial species under two distinct environmental conditions and documented the mode of bacterial growth. We also conducted experimental evolution with clonally derived Escherichia coli and documented the differences in the mode of bacterial growth before and after evolution.
Research sample	In the first part of our study, we used bacterial clones derived from laboratory stocks of different well-studied bacterial species. The rationale behind this choice of research sample was to test if the phenomenon of interest (phenotypically plastic cell clustering) can be exhibited by diverse bacteria (Gram-positive and Gram-negative). Next, we chose Escherichia coli K12 substr. MG1655 for conducting evolution experiments as it is a convenient and proven model system for such studies. We started our evolution experiments with a single clone because it facilitates genetic and phenotypic analyses of the ensuing evolution and is a standard practice in this area.
Sampling strategy	We used 5 independently evolving biological replicates for the evolution experiment, which is a standard practice in the subject area as this level of replication tends to be sufficient for capturing the biological variation in such experiments. Following another standard practice in the subject area, we sampled a clone randomly from each evolved replicate population (5 each for S and R, respectively) by streaking a random aliquot from well mixed vials.
Data collection	Clones were assayed by YC in an unbiased manner for growth patterns under two distinct environmental conditions at two different levels: 1. Populations growing in glass tubes (automated photography); 2. Single cells derived from populations (brightfield and fluorescence microscopy).
Timing and spatial scale	The experiment was done in the laboratory between January 2022 and August 2022. Spatial scale: The experiment cultured bacteria in 5 ml liquid medium within glass vials.
Data exclusions	No data were excluded.
Reproducibility	The study involved replicated assays over five independent biological replicates.
Randomization	Randomization is not applicable to our study because all the experiments were conducted in individual glass tubes and no tubes were excluded from the analyses.
Blinding	Blinding is not applicable to our study because all the samples within our replicates were treated together and equally.

Did the study involve field work? ☐ Yes ☒ No

Reporting for specific materials, systems and methods

We require information from authors about some types of materials, experimental systems and methods used in many studies. Here, indicate whether each material, system or method listed is relevant to your study. If you are not sure if a list item applies to your research, read the appropriate section before selecting a response.

Materials & experimental systems

n/a	Involved in the study
☒	☐ Antibodies
☒	☐ Eukaryotic cell lines
☒	☐ Palaeontology and archaeology
☒	☐ Animals and other organisms
☒	☐ Clinical data
☒	☐ Dual use research of concern
☒	☐ Plants

Methods

n/a	Involved in the study
☒	☐ ChIP-seq
☒	☐ Flow cytometry
☒	☐ MRI-based neuroimaging
