# Supplementary material for: Bacteria evolve macroscopic multicellularity by the genetic assimilation of phenotypically plastic cell clustering
Source: Nat Commun. 2023 Jun 15;14:3555. doi: 10.1038/s41467-023-39320-9 (PMC10272148; doi:10.1038/s41467-023-39320-9)
Supplement: Supplementary file 4 — Description of Additional Supplementary Files [file 41467_2023_39320_MOESM4_ESM.pdf]

**Description of Additional Supplementary Files**

Supplementary Data 1
Description: Mutations detected in the evolved S and R clones after experimental evolution

Supplementary Movie 1
Description: E. coli WT in habitual salinity

Supplementary Movie 2
Description: S. aureus in habitual salinity

Supplementary Movie 3
Description: E. coli WT in high salinity

Supplementary Movie 4
Description: S. aureus in high salinity

Supplementary Movie 5
Description: S clones in habitual salinity

Supplementary Movie 6
Description: R clones in habitual salinity

Supplementary Movie 7
Description: S clones in high salinity

Supplementary Movie 8
Description: R clones in high salinity

Supplementary Movie 9
Description: Clone S2 life cycle (high salinity)

Supplementary Movie 10
Description: Clone S5 life cycles (left: habitual salinity; right: high salinity)

Supplementary Movie 11
Description: Enzymatic assays with ancestor in high salinity (left: control; centre: cellulase; right: proteinase K)

Supplementary Movie 12
Description: Enzymatic assays with Clone S5 in high salinity (left: control; centre: cellulase; right: proteinase K)

Supplementary Movie 13
Description: Enzymatic assays with Clone S5 in habitual salinity (left: control; centre: cellulase; right: proteinase K)
